# The case for a national survey of eating disorders in Australia

**DOI:** 10.1186/s40337-018-0221-3

**Published:** 2018-10-15

**Authors:** L. M. Hart, D. Mitchison, P. J. Hay

**Affiliations:** 1 School of Psychology and Public Health, La Trobe University, Melbourne, Australia; 2 Melbourne School of Population and Global Health, University of Melbourne, Melbourne, Australia; 3 Department of Psychology, Centre for Emotional Health, Macquarie University, Sydney, Australia; 4 Translational Health Research Institute, School of Medicine, Western Sydney University, Sydney, Australia

## Abstract

In this Commentary we outline the case for a national survey of eating disorders in Australia. Given the recent focus of the federal government to provide further funding for mental health research, we call for a national survey to be made a key priority. Such high-quality, nationally representative data are critically important to informing all other domains of eating disorders research in the Australian context, and to informing the research agenda internationally.

Australia is in urgent need of a national epidemiological survey for eating disorders (EDs). To date, studies have been limited to specific populations (e.g., female twins [1], adolescents [2]) or States (e.g., South Australia (SA) [3]) and been inadequately powered to detect the full spectrum of EDs [2, 4]. Whilst anorexia nervosa (AN) and bulimia nervosa (BN) were included in the 1998 Australian Child and Adolescent Survey of Mental Health and Wellbeing [4], binge eating disorder (BED) and eating disorders not otherwise specified (i.e., the more prevalent eating disorders) were excluded [4]. EDs were excluded from the 2013–2014 replication of this adolescent national survey and have never been included in the adult Australian national mental health surveys. Overall this has had the effect of retaining a state of ignorance in terms of the burden of EDs within Australia.

Findings indicate that EDs are common. Hay and colleagues reported a total point-prevalence of any DSM-5 ED of 7.2% in SA adults in 2015 [3]. Likewise, in the Perth cohort study of 1,600 adolescents, Allen and colleagues reported point prevalence estimates for any DSM-5 ED of 1.2% and 8.5% in males and females at age 14, increasing to 2.9% and 15.2%, respectively, by age 20 [2]. ED symptoms are also on the rise in Australia. Mitchison and colleagues found the prevalence of at least weekly binge eating increased almost 5-fold over an 18-year period [5] with parallel increases not only in other behavioural and cognitive ED symptoms [6], but also in the comorbidity of these with obesity [7].

Internationally there are only four published national surveys (United States, Netherlands, New Zealand, Germany) [8–11], all of which were conducted prior to the DSM-5, and all but one limited to AN and BN. Given that DSM-5 brought in major diagnostic changes that are predicted to impact specific and total ED prevalence [2, 12], national surveys of DSM-5 EDs are needed.

So why has this not been achieved and what are the historical barriers? There is a widespread perception that EDs are uncommon, impacting only a small section of the general population – typically young, White females. This has perpetuated a cycle of misinformation. Historically, EDs were described as disorders of starvation or overeating that affected young females [13] and the earliest disorders to be included in the DSM systems were indeed those more common in younger women (i.e. AN and BN [14]). This characterisation led initial community-based studies to limit their surveys to AN and/or BN and young females and thus widespread underestimation of ED prevalence. Accumulated evidence and the eventual inclusion of BED into the DSM-5 has improved awareness, at least within the ED field, of EDs across genders, the lifespan, and weight status.

The widespread belief that EDs are rare also leads to their low priority in research funding, treatment trials and clinical care. Lack of funding and investigation then reinforces this belief. If it is thought that screening for EDs will offer up few positive cases, and only yield complex diagnoses that are difficult to treat, other disorders will be given priority. Further complicating the case for EDs is that symptoms are ego-syntonic [15] and are often positively rewarded whilst disordered eating behaviours are known to be a strong but maladaptive mechanism for emotional regulation. Thus EDs are often well advanced before treatment is considered [16]. The cumulative effect is that individuals with EDs seek help for co-morbidities (e.g., weight-loss or depression) but not their ED [17]. The lack of visibility of EDs – other than AN - in clinical care also serves to reinforce the notion that EDs are uncommon and low-priority. It also means that other methods of national data collection such as a registry would be a known underestimate because of the low rates of treatment seeking.

With national data, the burden of EDs can be both quantified and located. This information would facilitate targeted and early interventions [7]. At present, EDs are not routinely screened for in primary care settings [18]. This leads to poor management, chronicity, and a costly burden on tertiary health systems [19]. Further, help-seeking for these disorders is very low, at around 10% [17], even though many symptomatic individuals are in contact with health services [20]. Better understanding the hidden burden of EDs is also likely to lead to better outcomes for concomitant physical (e.g., obesity) and mental health (e.g., depression, anxiety, and substance abuse) outcomes [8].

In 1992 Henderson, wrote “*A National Survey was the only way to correct the deficiency [in prevalence data] if national policy was to be guided by sound data*” [21]. These sentiments have been echoed internationally. For example, the World Health Organisation global burden of disease study has been criticised for overreliance on poor quality data, even for “mainstream” disorders such as depression and anxiety [22]. Regarding the widely quoted depression estimates, one critical reanalysis of the quality of evidence found few countries had nationally representative data and concluded “*the uncritical application of these estimates to international health care policy-making could divert scarce resources from other public healthcare priorities*” [23] .

Despite neglect by Australian national mental health surveys [24, 25] AN is widely considered the mental illness with the highest mortality rate [26] and as many as 20% of those deaths attributable to suicide [27]. With an increased focus on suicide prevention among people with EDs [28], a new (2017–2022) national agenda for EDs is resulting in bi-partisan development of EDs policy [29], and new research funding is earmarked for EDs [30, 31].

## Conclusion

In conclusion we propose a comprehensive scientific program assessing the full spectrum of EDs and an economic analysis of their burden and the cost effectiveness of primary prevention options. The survey should be longitudinal which would be unique internationally and provide first time national-scale information in the same way that cancer registries currently operate [32, 33]. It would inform the current push to adequately fund EDs research and treatment services, lead to innovations in early intervention, detection and prevention, and provide an important impetus to include EDs in future national mental health surveys in other countries around the world.
